# Blood eosinophils in chronic obstructive pulmonary disease: A potential biomarker

**DOI:** 10.2478/jtim-2023-0096

**Published:** 2023-09-02

**Authors:** Yanan Cui, Yan Chen

**Affiliations:** Department of Pulmonary and Critical Care Medicine, The Second Xiangya Hospital of Central South University, Changsha 410011, Hunan Province, China

## Background

Chronic obstructive pulmonary disease (COPD) is a common, preventable and treatable disease that is characterized by persistent respiratory symptoms and airflow limitation.^[1]^ The underlying cellular and molecular mechanisms of COPD are still insufficiently understood. The variable clinical characteristics and differences in response to therapy suggest that COPD may have different phenotypes. The identification of phenotypes based on biomarkers could help clinicians to establish individual treatment programs for patients with COPD.

Eosinophils are key immunoeffector and inflammatory cells that have diverse functions, with roles in homeostasis and disease in various tissues.^[2]^ Evidence suggests that 20%–40% of stable COPD patients have airway eosinophilic inflammation,^[3]^ and 28% of acute exacerbations are associated with sputum eosinophilia.^[4]^ In China, it has been reported that eosinophilic airway inflammation is present in 38.04% of COPD patients with acute exacerbation,^[5]^ and more than 40% of the inpatients had a blood eosinophil count (BEC) ≥ 2%.^[6]^ The Global Initiative for Chronic Obstructive Lung Disease (GOLD) 2022 recommended BEC as a biomarker to estimate the benefits of using inhaled corticosteroids (ICS).^[1]^ However, current studies have produced conflicting results regarding the ability of blood eosinophils to predict clinical outcomes. Comprehensive data about the role of blood eosinophils in COPD is critical for clinicians. In the current study, we aim to give new insights about eosinophils in COPD based on recent evidence.

## Eosinophilic airway inflammation

**Figure 1:**
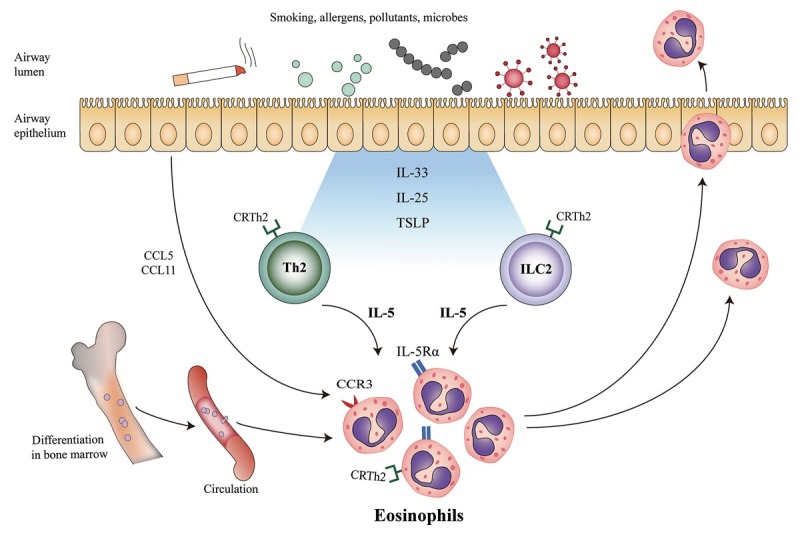
Eosinophilic airway inflammation in COPD. Th2: T helper-2 cells; CRTh2: chemoattractant receptor homologue from Th2 cells; ILC2: innate lymphoid cells; TSLP: thymic stromal lymphopoietin; IL: interleukin; CCL: C-C motif chemokine ligand; CCR: C-C motif chemokine receptor.

In COPD, although the major inflammatory cells are CD8^+^ T cells, neutrophils and macrophages, some patients have eosinophil involvement.^[7]^ Eosinophilic inflammation starts with the recruitment and migration of eosinophils through the blood circulation into the airway stimulated by the C-C motif chemokine ligand (CCL) 11 and CCL 5.^[8]^ Airway epithelial cells also release the upstream cytokines in response to noxious agents and recruit T helper-2 and innate lymphoid cells, which secrete IL-5, stimulating the maturation and release of eosinophils (Figure 1).^[7]^ Increased numbers of eosinophils have been reported in both the central and peripheral airways of patients with COPD, detected by bronchial biopsies, bronchoalveolar lavage or sputum.^[9]^ In 2021, Higham et al. reported a wider profile of T2 inflammation in eosinophilic COPD that extended to mechanisms including IL-13 driven pathways.^[10]^ This may provide a new therapeutic target for eosinophilic COPD.

## Stability of eosinophilia

The repeatability of BECs is essential for clinical application. A secondary analysis of the AERIS cohort including moderate-to-severe COPD patients showed that elevated BEC remained stable within individuals across the following year.^[11,12]^ Landis and colleagues also reported a goodreproducibility of BEC over one year in 27557 patients with stable COPD in general practice.^[13]^ Interestingly, Southworth and colleagues proposed that the greatest variation was observed in patients with BEC ≥ 300 cells/μL, and better reproducibility was observed at lower thresholds (100 or 150 cells/μL).^[14]^ In a recent prospective study, we assessed BECs on admission and discharge in patients hospitalized due to an exacerbation and found a discordance of 35.6% in patients admitted with a BEC ≥ 300 cells/μL, far exceeding the degree of inconsistency in patients with a low BEC at admission.^[15]^ Therefore, exploring the association between the variability of blood eosinophils and outcomes of COPD patients is more meaningful than investigating the value of a single BEC.

## Eosinophils and clinical characteristics

A few studies have indicated a possible link between eosinophil counts and clinical characteristics, such as symptoms, dyspnea score, lung function and bacterial load. The ECLIPSE cohort study reported that COPD patients with eosinophil counts persistently ≥ 2% were characterized by a higher forced expiratory volume in 1 s (FEV_1_) percentage predicted and fewer symptoms with lower dyspnea scores and lower BODE (body mass index, airflow obstruction, dyspnea, exercise capacity) index.^[16]^ Analyzing data from the ACURE study in China, we also found lower eosinophil level was associated with a higher risk of respiratory failure and higher mMRC dyspnea grade.^[6]^ In addition, a strong relationship between low levels of eosinophils and the development of pneumonia was reported in our study.^[6]^ There is an inverse relationship between eosinophil levels and bacterial infection with Streptococcus pneumoniae, Moraxella catarrhalis and Haemophilus influenzae during an exacerbation.^[17]^ Lower blood eosinophils increase the risk of pneumonia in patients with COPD treated with ICS.^[18]^ These results may be explained by the biological functions of eosinophils in the innate immune response against infections.

## Eosinophils and the response to corticosteroids

Studies have shown a compelling relationship between a high BEC and the greater effect of ICS in preventing future exacerbations. Sources of evidence include post-hoc analyses and pre-specified analyses. In the post-hoc analyses of FORWARD, TRISTAN and INSPIRE studies, significant differences in the rate of exacerbations were observed in patients receiving ICS compared with non-ICS therapy for those with high BEC at baseline.^[19,20]^ Data from three randomized controlled trials (RCTs) showed that, with an eosinophil count of 100 cells/μL or more, a significant treatment effect was recorded in terms of reducing exacerbations with budesonide-formoterol compared with formoterol alone.^[21]^ An analysis of the IMPACT trial showed the magnitude of benefit of treatments containing ICS in reducing rates of exacerbations increased in proportion with BEC.^[22]^ These findings have been verified in several RCTs^[23-25]^ and real-world clinical practice.^[26]^ The GOLD 2023 recently stated that for patients with frequent exacerbations, treatment should be initiated with a combination of long acting bronchodilators and ICS if the BEC ≥ 300 cells/μL.^[27]^

A few studies have analyzed the response to systemic corticosteroid therapy in patients with eosinophilia during COPD exacerbations. A multicenter, open-label, non-inferiority RCT in patients admitted to hospital with acute exacerbation of COPD (AECOPD) showed that the median duration of systemic corticosteroid therapy was lower in the eosinophil-guided group.^[28]^ In a prospective randomized trial, eosinophilic exacerbations were related to rapid symptomatic recovery and fewer treatment failures (defined as re-treatment, hospital admission or death) than non-eosinophilic exacerbations treated with systemic corticosteroids.^[29,30]^ A further analysis of three RCTs reached a similar conclusion.^[31]^ The ACURE study further confirmed that the hospitalization time following treatment with systemic corticosteroids was shorter in eosinophilic AECOPD compared to non-eosinophilic AECOPD in patients with a smoking history.^[6]^

## Eosinophils and clinical outcomes

Studies investigating the relationship between blood eosinophils and future exacerbation risk have showed different results. A large-scale, retrospective, observational cohort study with 34268 patients involved with a history of exacerbations found that BEC ≥ 150 cells/μL was associated with increased COPD-related emergency visits and higher exacerbation rate compared with BEC < 150 cells/μL.^[32]^ Patients with moderate-to-severe COPD with BEC ≥ 300 cells/μL had an increased risk of exacerbation in the COPDGene study, which was prospectively validated in the ECLIPSE study.^[33]^ A retrospective cohort study, in which most patients had no history of exacerbations, also proposed that high blood eosinophil levels were an independent risk factor for future exacerbations in COPD patients.^[34]^ In contrast to these results, the SPIROMICS cohort found no relationship between high BEC and COPD exacerbations.^[35]^ Furthermore, data from the CHAIN and BODE cohorts also showed that BEC ≥ 300 cells/μL persisting over 2 years was not a risk factor for COPD exacerbations.^[36]^ A post-hoc analysis pooled data from 11 Phase III and IV randomized COPD studies found no clinically important relationship between baseline BEC and exacerbation rate.^[37]^ The GOLD 2022 states that there is insufficient evidence to recommend blood eosinophils as a biomarker to predict exacerbation risk.^[1]^

Instead of using only a single BEC to evaluate prognosis, researchers should pay more attention to the association between the variability of BECs and clinical outcomes. A population-based study recently showed stable COPD patients with the highest variability in blood eosinophils more frequently experienced exacerbations.^[38,39]^ Scarce data exist exploring the stability of blood eosinophils during an AECOPD and its relationship to clinical outcomes. Recently, using two measurements at admission and discharge, we revealed that only patients with a persistently high BEC during admission had a higher risk of moderate-to-severe exacerbations.^[15]^ Meanwhile, we proposed that patients with a persistently low BEC tended to have poor survival.^[15]^ At least two BECs are required to identify patients with different outcomes.

## Perspectives

Eosinophilic COPD is associated with a profile of T2 inflammation. COPD patients with eosinophilic inflammation tend to have a lower airway bacterial load and respond better to corticosteroid treatment. Eosinophil changes could help to predict clinical outcomes of COPD patients. Further work is needed to understand the full clinical significances of blood eosinophils as a biomarker in COPD.
